# Role of *Lactobacillus* in Female Infertility *Via* Modulating Sperm Agglutination and Immobilization

**DOI:** 10.3389/fcimb.2020.620529

**Published:** 2021-01-25

**Authors:** Fenghao Zhang, Jie Dai, Tingtao Chen

**Affiliations:** ^1^ Institute of Translational Medicine & School of Life Sciences, Nanchang University, Nanchang, China; ^2^ Key Laboratory of Carcinogenesis and Translational Research (Ministry of Education/Beijing), Department of Renal Cancer and Melanoma, Peking University Cancer Hospital and Institute, Beijing, China

**Keywords:** vaginal microbiota, bacterial adherence, vaginal *Lactobacillus*, unexplained infertility, sperm agglutination

## Abstract

Infertility has become a common problem in recent decades. The pathogenesis of infertility is variable, but microbiological factors account for a large proportion of it. Dysbiosis of vaginal microbiota is reportedly associated with female infertility, but the influence of normal vaginal microbiota on infertility is unclear. In this review, we summarize the physiological characteristics of the vaginal tract and vaginal microbiota communities. We mainly focus on the bacterial adherence of vaginal *Lactobacillus* species. Given that the adherent effect plays a crucial role in the colonization of bacteria, we hypothesize that the adherent effect of vaginal *Lactobacillus* may also influence the fertility of the host. We also analyze the agglutination and immobilization effects of other bacteria, especially *Escherichia coli*, on ejaculated spermatozoa, and speculate on the possible effects of normal vaginal microbiota on female fertility.

## Introduction

Infertility has become a common problem in recent decades. Microbiological factors account for a large proportion of infertility, but attention has mainly focused on pathogenesis and infection caused by pathogens. With a further understanding of the host–microbe relationship, the interaction between the normal body microbiome and host cells has been considered important with regard to etiology. In females, vaginal microbiota plays a role in female infertility. The vagina has an intrinsic microbiota. Dysbiosis of the vaginal microbiota or invasion of pathogens can impair the fertility of humans by directly decreasing the motility and vitality of spermatozoa (Monga and Roberts, 1994; Sellami et al., 2014), or indirectly by inducing organic injuries of the reproductive system. However, influences from the normal vaginal microbiota on female fertility are unclear. *Lactobacillus* is the dominant bacterial genus in the vaginal tract. Evidence has shown that vaginal *Lactobacillus* play an essential role in preventing the invasion of foreign bacteria and dysbiosis of intrinsic microbiota, but its effect on ejaculated spermatozoa has been rarely reported. Bacterial adherence is an essential colonization process for vaginal *Lactobacillus*, and *Lactobacillus* species with strong adherent effects can provide more benefits to hosts. However, the same adherent effect may also modulate the chemical and physical properties of ejaculated spermatozoa. This phenomenon may account for some cases of unexplained infertility and provide a unique sperm selection mechanism for the human body.

## Infertility and Inflammation

Infertility is the condition of being unable to produce offspring. It is defined by the World Health Organization as ‘a disease of the reproductive system defined by the failure to achieve a clinical pregnancy after 12 months or more of regular unprotected sexual intercourse’ (Zegers-Hochschild et al., 2017). It affects about 8%–12% of couples worldwide; this prevalence has increased in recent decades (Kumar and Singh, 2015). Given that 95% of the world population considers becoming a parent as part of their adult development, infertility is a disruption to their life course (Bahamondes and Makuch, 2014). It also induces psychological suffering and discord in the marital relationship (Bahamondes and Makuch, 2014; Ahmadi Forooshany et al., 2014). In couples afflicted by infertility, 26%–30% of cases are caused by male factors, while 45%–60% are caused by female factors (Lindsay and Vitrikas, 2015). Male infertility is commonly due to deficiencies in sperm and semen. Male infertility pathogenesis includes inflammation and infection, injury and surgery, smoking, drinking, anatomic variance, genetic defect(s), immunological disorder, systemic disease and aging. Infection and inflammation of the urogenital tract account for more than 12% of male infertility (Dohle, 2003). *Chlamydia trachomatis* and *Neisseria gonorrhoeae* are the most common pathogens in sexually transmitted urogenital infection (Pellati et al., 2008), and uropathogenic *Escherichia coli* (UPEC) accounts for most cases of ascending urogenital infections (Pellati et al., 2008). Female infertility is mostly due to infections and inflammation such as salpingitis and vaginitis. *N. gonorrhoeae* and *C. trachomatis* are the most widely reported pathogens that lead to salpingitis. Other pathogens, such as *Mycoplasma genitalium* and *Trichomonas vaginalis*, have also been reported (Mitchell and Prabhu, 2013; Tsevat et al., 2017; Arustamyan et al., 2017). Bacterial vaginitis (BV) is the most prevalent vaginitis among women of reproductive age; approximately 40%–50% of vaginitis cases are BV (ACOG technical bulletin, 1996; Onderdonk et al., 2016). Vaginal candidiasis (20%–25%) and trichomoniasis (15%–20%) are the second and third most common vaginitis (ACOG technical bulletin, 1996). Notably, 10%–20% of infertility cases are unexplained (Lindsay and Vitrikas, 2015): the infertility does not have a clinical inflammation or related disease(s). Given that microbiological factors account for a large proportion of known infertility, it is rational to study the microbiological induced infertility under non-inflammatory conditions.

## Types and Probiotic Properties of the Vaginal Microbiome

It has long been known that the vagina contains its own microbiota. The interaction between host and the vaginal microbiota provides the unique microenvironment of the vaginal tract.

### *Lactobacillus*: Dominant Genus of the Vaginal Microbiome Under Physiological Conditions

The profile of the vaginal microbiome differs depending on age, but for most cases of healthy women of reproductive age, *Lactobacillus* species are the dominant vaginal bacteria (Martin and Marrazzo, 2016; Godha et al., 2017). *Lactobacillus* in the vagina is highly limited to certain strains (Nunn and Forney, 2016). In most cases, *Lactobacillus crispatus*, *Lactobacillus gasseri*, *Lactobacillus jensenii* and *Lactobacillus iners* dominate the vaginal microbiome (Nunn and Forney, 2016). One of the important factors that influence the profile of vaginal microbiota is the concentration of local estrogen. A high estrogen concentration can induce a thicker vagina mucosa, upregulate proton secretion by vaginal epithelial cells and accelerate the deposition of glycogen in the vaginal epithelium (Semmens and Wagner, 1982; Gorodeski et al., 2005; Godha et al., 2017). All these changes enrich the vaginal microbiota, especially *Lactobacillus* species. The pH level of the vaginal cavity maintains a dynamic stability throughout the reproductive age, and accumulation of lactic acid released by *Lactobacillus* species plays a major role on the acidification process.

### Profile and Community State Types of the Vaginal Microbiome

In terms of the dominant bacterial species, vaginal microbiota can be divided into five community state types (CSTs). The microbiome dominated by *L. crispatus*, *L. gasseri*, *L. iners*, and *L. jensenii* is separately classified as CST I, II, III, and V, respectively (Ravel et al., 2011; Nunn and Forney, 2016). Cases that are dominated by more than one *Lactobacillus* species are very rare in black women but common in Caucasian and Asian women (Zhou et al., 2010). The final community type (CST IV) is dominated by other anaerobic bacteria such as *Gardnerella vaginalis*, *Prevotella*, *Atopobium*, and *Megasphaera* instead of *Lactobacillus* (Ravel et al., 2011). CST IV can be further divided into CST IV-A and CST IV-B. CST IV-A does not have an obvious dominant bacterial genus and is composed of low proportions of *Lactobacillus* species and other anaerobic species such as *Anaerococcus, Corynebacterium, Finegoldia*, and *Streptococcus*, while CST IV-B is dominated by *Atopobium* and accompanied by species from *Prevotella, Parvimonas, Sneathia, Gardnerella, Mobiluncus*, or *Peptoniphilus* (Gajer et al., 2012). Many bacterial species present in CST IV are considered to be the pathogens of BV. Thus, the presence of CST IV may indicate a subclinical BV, and CST IV-B is more likely to develop into a clinical infection due to its low ratio of *Lactobacillus* colonization. Racial differences in vaginal microbiota have been reported. Fettweis et al. showed that white women were more likely to develop a *Lactobacillus*-dominant vaginal microbiota compared to black women (Fettweis et al., 2014). Zhou et al. showed that Japanese and Caucasian women were more likely to develop a vaginal microbiota dominated by multiple *Lactobacillus* species compared with black women (Zhou et al., 2010). As a result, black women are more likely to develop a vaginal microbiome with a lower proportion of *Lactobacillus* species.

### Probiotic Effect of Vaginal Lactobacilli

Vaginal lactobacilli play an essential role in maintaining a healthy female genital system. The protective benefits of vaginal *Lactobacillus* have been widely reported. These protective benefits can be attributed to bactericidal substances, ecological niche occupation and immunomodulatory effects. Therein, lactic acid secreted by vaginal lactobacilli plays a major role. The protective benefits of lactic acid have been widely reported. Some studies have shown that a physiological concentration of lactic acid in the vagina can effectively inactivate HIV and inhibit the growth of uropathogenic bacteria and BV-associated pathogens (Aldunate et al., 2013a; Gong et al., 2014; Leccese Terraf et al., 2017). Direct inactivation is the major effect responsible for this benefit (Aldunate et al., 2013b), and the immunomodulatory effect plays a controversial role in this process (Witkin et al., 2011; Hearps et al., 2017). Hydrogen peroxide (H_2_O_2_) had been described as the main bactericidal substance produced by vaginal *Lactobacillus*, but recent studies have contradicted the effect of H_2_O_2_ (Eschenbach et al., 1989; Mitchell et al., 2015; Tomás et al., 2016; Tachedjian et al., 2018). Hence, the acid-producing abilities of *Lactobacillus* species have become the major criterion when evaluating their probiotic benefits. Based on an *in vitro* test, *L. crispatus* accumulates a higher lactic acid concentration compared with *L. iners* (Witkin et al., 2013). Thus, *L. crispatus* has a more potent probiotic effect compared with *L. iners*. Vaginal *Lactobacillus* species also prevent the occurrence and development of cancer cells. In several studies, patients with cervical intraepithelial neoplasia were more likely to have a low concentration of vaginal *Lactobacillus* species (Mitra et al., 2016). Other authors have demonstrated the anti-tumor effect of *L. gasseri*; this effect is mediated by directly inhibiting the proliferation of tumor cells (Wang et al., 2017), or indirectly by promoting the clearance of human papilloma virus (HPV) (Brotman et al., 2014). Notably, the inhibitory effect of *L. gasseri* on tumor cells is independent of pH level and lactate (Motevaseli et al., 2013); exopolysaccharides (EPSs) produced by *Lactobacillus* may play an essential role in this process (Sungur et al., 2017). The protective benefit against other pathogens and inflammation also reduces the risk of cervical cancers, and similar benefit can also be observed in other gynecological cancers (Ghosh et al., 2016; Ramchander and Crosbie, 2018).

### Dysbiosis of the Vaginal Microbiome Indicates a Vulnerable Condition

Dysbiosis of the vaginal microbiota often presents as BV. The major characteristic of BV is the shift from a *Lactobacillus*-dominant flora to a polymicrobial flora in the vaginal tract (Onderdonk et al., 2016). Unlike other forms of vaginitis, BV is a complex syndrome that is not caused by a specific pathogen. This indeterminate pathogenicity makes it difficult to diagnose *via* a single criterion. To date, the Nugent score and Amsel criteria are two commonly used measures to evaluate BV (Kenyon and Osbak, 2014). The Nugent score focuses on the morphotypes of bacteria in vaginal smear (Nugent et al., 1991), while Amsel criteria focus on the physical characteristics of vaginal swabs (Amsel et al., 1983; Kenyon and Osbak, 2014). BV has a strong association with infertility (Salah et al., 2013; Babu et al., 2017). Ascending infection of BV-associated bacteria will impair the function and immunity barrier of the upper genital system (Racicot et al., 2013; Ravel and Brotman, 2016). In addition, there has been a reported direct impairment effect of BV-associated pathogens on ejaculated spermatozoa. This phenomenon provides some hints about the interaction between the vaginal microbiome and ejaculated spermatozoa.

## Types and Pathogenic Properties of the Semen Microbiome

The semen of healthy males had been considered a sterile condition because traditional bacterial cultivation provided an incomplete characterization of the full semen microbiome profile. With the development of high-throughput sequencing, insights into the semen microbiome have been gradually revealed, but the available studies are still insufficient.

### Uropathogens, Semen Quality, and Dysspermia

Based on recent studies, most normal semen contains bacteria, and 30% of normal semen samples contain overt bacteria that can be cultivated (Vilvanathan et al., 2016; Zeyad et al., 2018). The inflammation and infection caused by uropathogens play an essential role in infertility. Most common uropathogens are associated with dysspermia: they can influence the semen quality in spermatogenesis, reservation and fertilization (Farsimadan and Motamedifar, 2020). Some uropathogens can directly impair the performance of spermatozoa (**Table 1**), but the majority of uropathogens impair the semen quality during spermatogenesis. However, the spermicidal effect is evaluated *in vitro*, and this approach may differ from the physiological state. Although the virulent factors of many common uropathogens have been identified, it remains controversial whether the presence of these uropathogens can impair the semen quality. Some studies have shown that the presence of these pathogens is inconsistence with the abnormal semen parameters (Filipiak et al., 2015; Vilvanathan et al., 2016). Given that a mature male can release billions of activatable spermatozoa in one mate, asymptomatic bacteriospermia with a low bacterial load would likely not cause a prominent decrease in semen quality (Vilvanathan et al., 2016). The bactericidal molecules in semen also inhibit the bacterial spermicidal effect (Schulze et al., 2020).

**Table 1:** Agglutination and immobilization factors of some pathogens.

Impairment model	Year	Bacteria	Thermostability		Isolated source	Adherent site	Molecule
Agglutination factor	2009	*E coil* (Prabha et al., 2009b)	Labile	Cell body		Head, neck and tail	71 KDa protein
	2014	*E coil* (Kaur and Prabha, 2014)	Labile	Cell body		Head and tail	unknown
	1993	*E coil* (Wolff et al., 1993)	Labile	Cell body		Head and tail	Molecule in type 1 pili
	2013	*E coil* (Aucky Hinting DMS, 2013)	Stable	Cell body		Head, neck and tail	32.2 KDa protein
	2005	*S aureus* (Ohri and Prabha, 2005)	Labile	Cell body		tail	70 KDa protein
	2019	*S warneri* (Pant et al., 2019)	Unknown	Cell body		Head, neck and tail	80 KDa protein
Immobilization factor	2010	*E coil* (Prabha et al., 2010; Kumar et al., 2011)	Labile	Supernant			56 KDa protein
	1977	*E coil* (Paulson and Polakoski, 1977)	Stable	Supernant			<500 mw Small molecule
	2009	*Streptococcus* (Prabha et al., 2009a)	Labile	Supernant			~20 KDa protein
	2007	*Enterococcus* (Qiang et al., 2007)	Unknown	Cell body			β-hemolysin

### Seminal *Lactobacillus*, Community Types, and Dominant Species

When considering the prevalence of bacterial species in the semen microbiome in healthy males, it is essential to detail the profile of the semen microbiome. The significance of semen bacterial communities remains to be investigated; in earlier studies, researchers had been inclined to consider asymptomatic bacteriospermia as a pre-inflammatory condition, while in recent studies, researchers have considered some uropathogens as part of the normal flora in semen (Farsimadan and Motamedifar, 2020). Farahani et al. summarized the recent studies on the semen microbiome using high-throughput sequencing (Farahani et al., 2020). The authors showed that some common uropathogens such as *Ureaplasma urealyticum*, *Enterococcus faecalis* and *Mycoplasma hominis* have strong associations with male infertility, while other uropathogens, such as *C. trachomatis*, *E. coli*, and *Staphylococcus aureus* are not associated with low semen quality even though their spermicidal effects have been widely reported (Farahani et al., 2020). Semen communities reported in different studies are disparate, and their influence on semen quality has been controversial. Monteiro et al. revealed that *Enterococcus* was a dominant genus among 118 samples of normal semen, low-sperm-concentration semen, low-sperm-motility semen and abnormal morphylogy semen, and *Lactobacillus* represented a very low percentage of the semen microbiota in all samples (0.5%) (Monteiro et al., 2018). Hou et al. separated the semen microbiota from 77 samples with six CSTs according to the Calinski–Harabasz index. CST IV and V were predominated by *Ralstonia* and *Lactobacillus* while other CSTs were characterized by a variety of bacteria without a dominant genus. Semen from infertile patients had no significant differences in CSTs compared with normal samples, which indicates that the probiotic effect of *Lactobacillus* in semen quality is limited (Hou et al., 2013). Another two studies have highlighted the probiotic effect of seminal *Lactobacillus*. Weng et al. separated the semen microbiome from 96 samples into three CSTs, which were predominated by *Pseudomonas*, *Lactobacillus* and *Prevotella*, respectively. Most samples with normal semen quality were dominated by *Lactobacillus* (Weng et al., 2014). Baud et al. separated semen microbiome from 94 samples into three CSTs, which were characterized by high levels of *Prevotella* and *Lactobacillus* and a balanced representation of *Corynebacterium*, *Staphylococcus*, and *Planococcaceae*. Samples with normal sperm morphology had a higher level of *Lactobacillus*, but samples with low sperm motility and vitality had the same level of *Lactobacillus* compared with counterparts (Baud et al., 2019). Generally, a relatively high percentage of seminal *Lactobacillus* is positively associated with semen quality, which indicates *Lactobacillus* species may play a probiotic role on male genial tract.

#### Uropathogenic Escherichia coli, Sperm Agglutination and Immobilization

In previous studies, researchers have mainly focused on the inflammatory response caused by an infection instead of the direct spermicidal effect of pathogens. UPEC is the most widely reported uropathogen, and it presents a direct spermicidal effect. As early as 1931, Rosenthal had demonstrated an agglutination effect of *E. coli* on spermatozoa, and subsequent studies have also proven its immobilization effect on spermatozoa. Various proteins and molecules that contribute to these effects have been isolated. Fimbriae are the major structure responsible for the agglutination effect of *E. coli*. Two kinds of fimbriae are present on the surface of *E. coli*: type 1 pili and type P pili (Monga and Roberts, 1994). Type 1 pili can recognize the α-D mannose group on the head of spermatozoa, while type P pili can recognize the a-D-galp-l-4-9-D-galp group on the tail of spermatozoa. Both type 1 and type P fimbriae present a hemophilic adhesion effect; hence, bacteria that contain these two fimbriae produce an agglutination effect on spermatozoa. Different bacteria or even different strains of *E. coli* show broad diversity on the categories and numbers of fimbriae. Bartoov et al. indicated that type 1 pili might play a major role in urogenital infection (Bartoov et al., 1991). Aucky et al. isolated a hemagglutinin protein (32.2 kDa) on the terminal of fimbriae, which might be responsible for the adhesion of *E. coli* to spermatozoa (Aucky Hinting DMS, 2013).

Despite the sperm adherent effect, the direct spermicidal effect of *E. coli* has been adequately detailed. Sperm immobilization is a sophisticated process that can be disrupted by a variety of factors. Some studies have demonstrated the effect of lipopolysaccharide (LPS) on this process; LPS is a common endotoxin present on the surface of *E. coli* that can bind to Toll-like receptor 4 (TLR4), present on the membrane of spermatozoa, and consequently decrease sperm motility (Fujita et al., 2011). The possible mechanism of this impairment is that activation of the TLR signaling cascade increases the reactive oxygen species (ROS) level and therefore disrupts the membrane conformation of spermatozoa. This disruption can be hindered by ROS scavengers such as superoxide dismutase (SOD) (Urata et al., 2001; Fujita et al., 2011). Exotoxins also play a role in this process. A common *E. coli* exotoxin, hemolysin, can enhance the sperm immobilization mediated by pathogenic *E. coli* (Boguen et al., 2015). Hemolytic *E. coli* strains reportedly immobilize spermatozoa at a lower concentration compared with non-hemolytic strains. They can also induce a higher intracellular ROS level and lower sperm mitochondrial membrane potential (Δψm) (Boguen et al., 2015). α-Hemolysin (HlyA) is the most common reported hemolysin in UPEC; this 110 kDa protein contains several toxin family repeats (Wiles and Mulvey, 2013). HlyA can insert into the membrane of some cell types and assemble into a transmembrane pore. Studies have demonstrated that various receptors may mediate this transmembrane effect, for example, LFA-1 on the surface of leucocytes, but the transmembrane effect is generally considered to be non-specific (Wiles and Mulvey, 2013). Notably, α-HlyA of *E. coli* is transported by a type I secretion mechanism, and this mechanism transports hemolysin only when the bacteria directly contacts the cells (Kim et al., 2008). Hence, these immobilization factors are isolated from the bacterial body and only exert an effect when the bacteria adhere to the sperm. In addition to bacterial bodies, sperm immobilization can also be observed in supernatant or filtrate of a bacterial suspension. An early study reported a small soluble sperm immobilization factor (SIF) – a heat-stable small molecule – in an *E. coli* filtrate (Paulson and Polakoski, 1977). In recent studies, researchers have reported that *E. coli* contains a large SIF, a heat-labile, 56 kDa protein that can recognize a specific 113 KDa receptor presented on the membrane of spermatozoa (Prabha et al., 2010; Kumar et al., 2011). This SIF has a significant inhibitory effect on Mg^2+^-dependent ATPase activity and acrosome reaction induced by calcium ionophore (Vander et al., 2013).

### Spermicidal Effect of Other Pathogens

In studies of sperm agglutination and immobilization effects in other bacteria, researchers have also isolated some related molecules. Prabha et al. isolated a 20 KDa SIF from *S. aureus* filtrates; this heat-labile protein can recognize a specific 62 kDa receptor on the surface of spermatozoa (Prabha et al., 2009a). It inhibits Mg^2+^-dependent ATPase activity and the acrosome reaction to a lesser extent compared with *E. coli* (Gupta and Prabha, 2012). These data indicate that the Mg^2+^-dependent ATPase plays an important role on sperm motility function. Pant et al. isolated an 80 kDa sperm agglutination factor from *Staphylococcus warneri*; it also inhibits Mg^2+^-dependent ATPase activity and showed a potent contraceptive effect in mouse models (Pant et al., 2019). Ohri et al. isolated a 70 kDa protein from the cell culture of *S. aureus*; this protein mediates a tail-to-tail agglutination of spermatozoa (Ohri and Prabha, 2005). β-Hemolysin isolated from *Enterococcus* also impairs sperm motility, similar to HlyA; it impacts the membrane integrity and thus contributes to its toxic effect (Qiang et al., 2007). Sperm agglutination and immobilization have been shown for other pathogens, such as *C. trachomatis*, *Mycoplasma* species and *T. vaginalis* (Monga and Roberts, 1994; Sellami et al., 2014). The agglutination and immobilization effects of bacteria on human sperm are simultaneous and reversible in most cases, but high concentrations of some factors, such as hemolysin and LPS, can remarkably reduce the vitality of spermatozoa. Agglutinated sperm often show disruption in membrane morphology. An elevated ROS level and reduced Δψm are associated with necrosis and apoptosis of spermatozoa. In conclusion, the sperm impairment effect of bacteria is multifactorial and mediated by a variety of mechanisms.

## Roles of Lactobacillus on Fertility

The probiotic effect of *Lactobacillus* on the male and female genital tract have been discussed above. Given that the *Lactobacillus* presents a probiotic effect on both gametes and the microenvironment, it is easy to assume that *Lactobacillus* also plays a positive role on fertility. However, fertilization is a dynamic process that requires the gametes maintaining vitality in several microenvironments, a factor that is usually ignored during *in vitro* studies.

### Bacterial Concentration and the Adherent Effect

For years, vaginal *Lactobacilli* had been considered as totally beneficial components of the genital system, while the seminal *Lactobacillus* were still controversial. An essential point that had been ignored in the previous studies is that the concentrations of vaginal *Lactobacillus* and seminal *Lactobacillus* are totally different. Ejaculated spermatozoa stay in the vaginal tract for a period before fertilization. Even if seminal *Lactobacillus* have a positive effect on spermatozoa, it remains dubious whether vaginal *Lactobacillus*, which are far higher in concentration, exert a positive effect on ejaculated spermatozoa.

The adherent ability is a crucial property; it varies with the alteration of bacterial concentration. The adherent ability is an important criterion for evaluating the probiotic or pathogenic effect of microbes. Bacterial adhesion is the first step in colonization. It determines the invasive ability of pathogens and the potential of probiotics. The adherent effect of vaginal *Lactobacillus* species has been extensively studied. Species with a strong adherent effect are considered to be more beneficial to the human body, and probiotics with higher adherence to cells or the extracellular matrix, such as *Lactobacillus rhamnosus* and *Lactobacillus fermentum*, are more frequently used for clinical treatment (Shokryazdan et al., 2014; Verdenelli et al., 2014; Homayouni et al., 2014).

**Figure 1:**
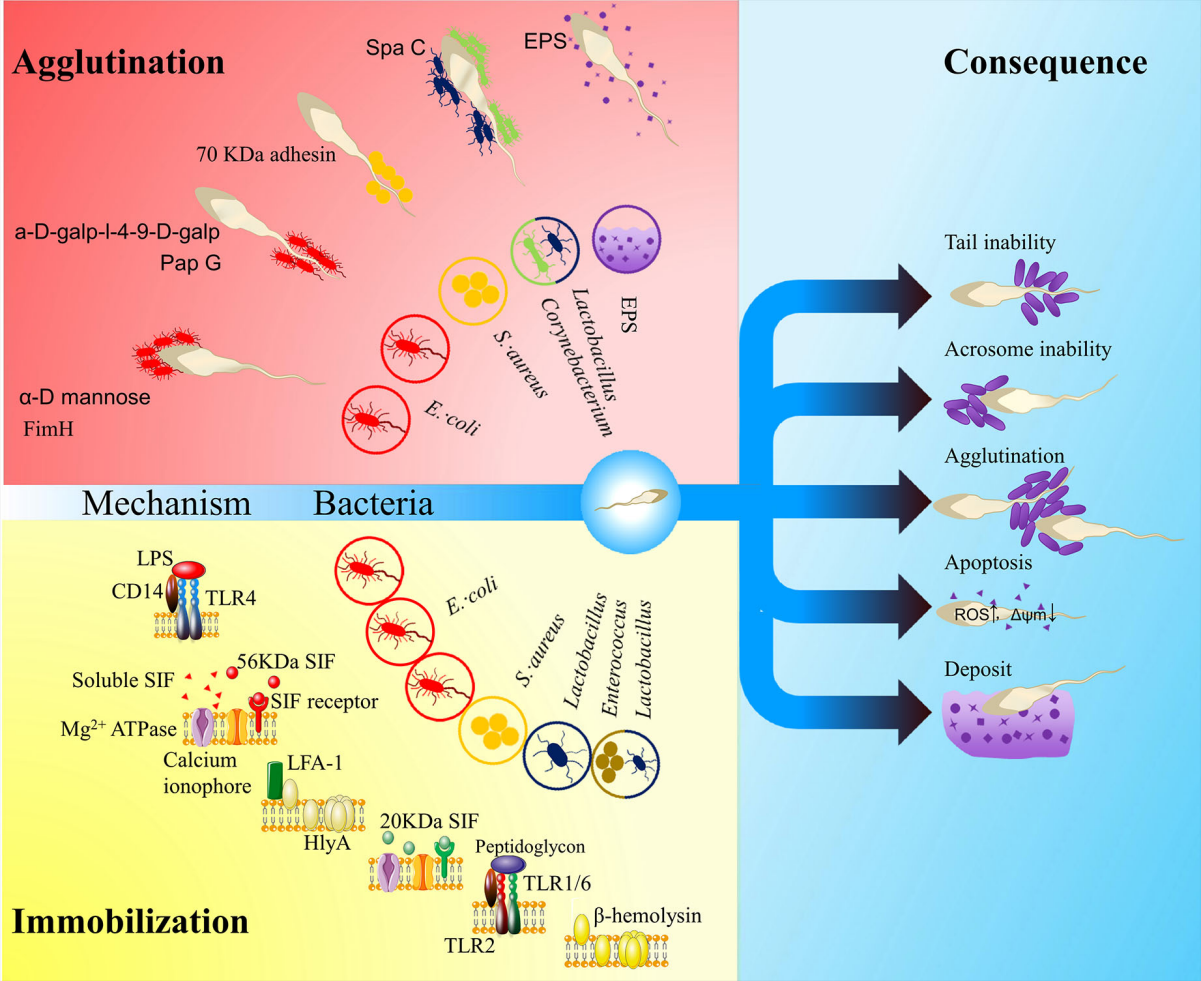
The influence of vaginal bacteria on ejaculated spermatozoa. The red area shows agglutination models: FimA located along the type 1 pilus of *Escherichia coli* binds to α-D mannose; PapG located at the tip of type P pilus binds to a-D-galp-l-4-9-D-galp (Monga and Roberts, 1994); a kind of 70 kDa adhesin on the surface of *Staphylococcus aureus* mediates sperm agglutination effect (Ohri and Prabha, 2005); SpaC located along the pilus of the *Corynebacterium* and *Lactobacillus* mediate the sperm agglutination effect (Makarova et al., 2006; Barbonetti et al., 2013); exopolysaccharides (EPSs) of the biofilm may also impact the function of spermatozoa. The yellow area shows immobilization models: lipopolysaccharide (LPS) in *E. coli* recognizes Toll-like receptor 4 (TLR4) (Fujita et al., 2011); a soluble small molecule mediates the sperm immobilization effect (Paulson and Polakoski, 1977); a 56 kDa sperm immobilization factor (SIF) recognizes a 113 kDa receptor and thereby impacts Mg^2+^-dependent ATPase and calcium ionophore (Prabha et al., 2010); α-hemolysin (HlyA) in *E. coli* impacts the membrane integrity of spermatozoa by LFA-1-mediated insertion or non-specific insertion (Wiles and Mulvey, 2013); a 20 KDa SIF from *S. aureus* can recognize a 62KDa receptor, which impact the Mg^2+^-dependent ATPase and calcium ionophore (Prabha et al., 2009a; Prabha et al., 2009b; Gupta and Prabha, 2012); *Lactobacillus* peptidoglycan recognizes TLR2 (Fujita et al., 2011); β- hemolysin from *Enterococcus* or perhaps *Lactobacillus* also impacts the membrane integrity of spermatozoa (Qiang et al., 2007). The blue area shows consequences: irremovability, acrosome impairment, agglutination, apoptosis and deposit. The hollow circles show the related bacteria or matrix.

With a high concentration, the adherence of vaginal *Lactobacillus* to spermatozoa may differ from that of seminal *Lactobacillus.* Ejaculated spermatozoa are mobile planktonic cells. Given that spermatozoa motility is a critical criterion for evaluating fertility, bacterial adherence may increase the load of spermatozoa and therein impair the motility of spermatozoa. Spermatozoa with lower motility have more chances to become adherent, and the binding of bacteria increases the load of cells and in turn reduces their mobility. With high concentrations of bacteria in the vaginal tract, these spermatozoa may be deposited and lose their vitality. Bacteria that adhere to spermatozoa at some specific sites may induce negative effects. Bacteria that adhere to the acrosome of the spermatozoa may block critical fertilization mechanisms. Hence, species with an adhesion preference to the acrosome may present a strong negative effect. Immobilized spermatozoa can act as a kernel that attracts planktonic bacteria to form complexes. When the concentration of planktonic bacteria reaches a threshold, numerous complexes may agglutinate and form a huge net structure that may intercept the swimming of normal spermatozoa. Widespread agglutination of bacterial bodies may induce the secretion of EPSs and initiate biofilm formation. Despite the direct attachment, the release of some exotoxins may immobilize spermatozoa and impair their fertilization ability. All these negative effects require a high bacterial concentration, which is unusual in semen but common in vaginal tract (**Figure 1**).

### Adherent Properties of *Lactobacillus* to Spermatozoa

The adherence of *Lactobacillus* to spermatozoa has been rarely reported. In a recent *in vitro* study, vaginal *Lactobacillus* strains presented a far stronger adherent effect on spermatozoa compared with *Enterococcus*, *Bacteroides*, *Bifidobacterium* and *Enterobacteriaceae* (Wang et al., 2019). In a co-incubation test of single bacterial species and ejaculated spermatozoa, this adherent effect significantly impaired the motility of spermatozoa (Wang et al., 2019).

The mechanism of bacterial adherence is complicated. In general, pili, afimbrial adhesins and interfacial free energy may play a role in this process. pili are hair-like appendage on the surface of bacteria. SpaCBA and LrpCBA are two major types of pili observed on the surface of some *Lactobacillus* species. SpaCBA is a kind of common pilus observed in varieties of Gram-positive bacteria. It plays a role on colonization by binding to host epithelial cells, mucin, mucous collagen as well as inducing bacterial aggregation. This pilus is observed in seminal *Corynebacterium*, and these pili may be associated with an exclusive sperm impairment effect on sperm motility without impacting the morphology and vitality of these cells (Turk et al., 2007; Mashaly et al., 2016). LrpCBA can be observed in most strains of *L. ruminis*. This pilus has a trend to bind type I collagen, fibronectin and host epithelial cells but lacks the ability of homophilic interaction. Some of *Lactobacillus* species *(L. casei, L. paracasei* and *L. rhamnosus*) contain the operon of anther pilus called SpaFED, recombinant SpaFED shows a similar adhesive property with SpaCBA, but this pilus lacks expression in native condition (**Table 2**) (Segers and Lebeer, 2014; von Ossowski, 2017). A putative mannose binding lectin gene is identified in the genome of *L. plantarum*. Given the mannose binding lectins of *E. coli* type 1 pili mediate the sperm adherent effect of UPEC, this gene may mediate the sperm adherent effect of *Lactobacillus* speices (Malik et al., 2016). In addition, the long fibrous structure of a pilus also contributes to the adherent process. Given that the strength of interfacial free energy is associated with the surface area, a pilus can easily pierce the energy barrier between two surfaces due to its small radii (Hori and Matsumoto, 2010).

**Table 2:** Fimbriae and microbial surface components recognizing adhesive matrix molecules (MSCRAMMs) of *Lactobacillus* species.

		Binding sites	Expression in lactobacilli	Expression in uropathogens
Fimbriae	SpaCBA (von Ossowski, 2017)	intestinal epithelial cells mucin type I/IV collagen SpaCBA pili	L. casei, L. paracasei, L. rhamnosus	Enterococcus faecium Enterococcus faecalis
	LrpCBA (von Ossowski, 2017)	intestinal epithelial cells type I collagen fibronectin	L. ruminis	absent
	SpaFED (Rintahaka et al., 2014)	intestinal epithelial cells mucin type I/IV collagen fibronectin	absent	absent
MSCRAMMs	Cna (Herman-Bausier et al., 2016)	type II Collagen	L. salivarius L. animalis L. casei L. plantarum	Staphylococcusaureus
	SdrC (Barbu et al., 2014)	SdrC	L. gasseri L. acidophilus L. paragasseri L. casei L. animalis	Staphylococcus aureus

Afimbrial adhesins also play an essential role in bacterial adhesion. A group of proteins called microbial surface components recognizing adhesive matrix molecules (MSCRAMMs), have been identified in some gram-positive bacteria. These molecules covalently link to peptidoglycans in the cell wall by sortases and target proteins in the host’s extracellular matrix (Vengadesan and Narayana, 2011). Cna and SdrC are two kinds of MSCRAMMs detected on the *Lactobacillus* species (Barbu et al., 2014; Chahales and Thanassi, 2015). These molecules are originally detected on the surface of Staphylococcus aureus. Cna plays a role on bacterial colonization by bind to collagen II of extracellular matrix (Herman-Bausier et al., 2016). SdrC plays a role on bacteria aggregation by a mild homophilic interaction. It also induces a strong affinity to some hydrophobic substances (**Table 2**) (Barbu et al., 2014). Numerous adhesins of the cell surface have been derived from gene sharing, a phenomenon where one protein can perform multiple functions in unrelated biological processes (Jeffery, 2003). The proteins that present gene sharing are called moonlighter proteins. Some enzymes, such as glyceraldehyde-3-phosphate dehydrogenase, L-lactate dehydrogenase, phosphoglyceromutase and UTP-glucose-1-phosphate, are used as moonlighter proteins in bacteria (Celebioglu and Svensson, 2018). They are secreted to the cell surface and act as adhesins, despite their roles in metabolic pathways. Moonlight proteins are detected on the surface of many *Lactobacillus species* (Sengupta et al., 2013), Borgdorff H et al. isolated some of moonlight proteins of *L. iners* and *L. crispatus*, their studies indicated that these proteins provide extra colonization benefits by playing a role of adhesins (Borgdorff et al., 2016).

Physical characters of bacterial cells also influence adherence. Charge distribution, hydrophobicity and the area of the contact surface are major physical factors that affect bacterial adherence. The influence of these factors can be quantified as the interfacial free energy. Hori demonstrated how to calculate the interfacial free energy and how the interfacial free energy influences the adhesion process (Hori and Matsumoto, 2010). This theory partly explains the influence of solution pH and hydrophobicity of cell surface substrates on adherence. Adhesion is favored when free energy is negative, and positive free energy provides an energy barrier between two surfaces and disturbs the adherence (Hori and Matsumoto, 2010). Interfacial free energy mainly influences the initial phase of adherence, in which the bacteria attach to a surface and form a transient, reversible and non-specific adherence. This initial adherence allows adhesins to bind to the surface, and then the bacterial adhesins will lead to an irreversible time-dependent adhesion after the initial phase (Katsikogianni and Missirlis, 2004). Some allosteric proteins such as acetolactate synthase will undergo a force-induced unfolding during the initial phase. The unfolding process alters their hydrophobicity and initiates a firm hydrophobic adhesion (Dufrêne, 2015). This strategy is reported in yeast cells, but the Als family of adhesins is also expressed in some *Lactobacillus* species (Siezen et al., 2012).

### Host Factors That Influence Bacterial Adherence

Under physiological conditions, the adherent pattern of bacteria is much more complex. Bacteria in the human body adhere to host epithelial cells, mucus and other bacteria. The interaction between bacteria and mucus plays a specific role on bacterial colonization. Mucins (MUC) are the major mucus substrate that interact with colonized bacteria. They are macromolecular glycoproteins that are secreted by specialized epithelial cells. In the female genital tract, mucosal epithelial cells express the transmembrane mucins MUC1, MUC4 and MUC16 on the cell surface, and goblet cells secrete mucus-forming mucins MUC5AC, MUC5B and MUC6 to the outer environment (Taherali et al., 2018). Transmembrane mucins provide adherent sites for vaginal bacteria so that they facilitate the colonization of vaginal bacteria. However, the effect of mucus-forming mucins on bacterial colonization is ambiguous. Free mucins provide adherent sites and nutrition for vaginal *Lactobacillus* species in a static condition, but a high level of free mucins will cover the surface of bacteria and disturb the attachment. Thus, planktonic bacteria will finally be washed out by vaginal fluid. The major mucin component of vaginal mucus is MUC5B (Portal et al., 2017; Taherali et al., 2018), and the secretion of MUC5B in the vaginal tract fluctuates during the menstrual cycle (Gipson et al., 2001); hence, the properties of mucus on bacterial adhesion may be different at different periods. The major ligands for bacterial adhesins of mucins are O-glycans (Sicard et al., 2017). O-glycan deficiency or lack of secretion alters vaginal bacterial communities (Ringot-Destrez et al., 2017). At the ovulatory phase, mucins with a neutral oligosaccharides on O-glycans are abundant compared with the acidic oligosaccharides at the follicular phase (Taherali et al., 2018). In addition to mucins, other matrix proteins such as fibronectin, fibrinogen and vitronectin also act as adherent sites.

### State of Aggregation and Bacterial Adherence

Bacteria can also attach to each other, a process known as agglutination. Bacteria with a high agglutination potential can form a complex architecture called a biofilm (Toyofuku et al., 2016). Biofilm formation provides a more hospitable outer environment for bacterial colonization. Intricate host–bacteria and bacteria–bacteria adhesions allow these bacteria to firmly occupy niches. A thick EPS layer limits the diffusion of antimicrobial agents, a factor that helps the bacteria evade the host immune system (Rabin et al., 2015). Besides, the high density of bacterial communities facilitates DNA exchange and therein exacerbates the spread of drug resistance (Rabin et al., 2015). Biofilm formation seems to be a natural – and sometimes – predominant step for colonization (Rabin et al., 2015). Planktonic bacteria first attach and adhere to a surface, and then colonized bacteria secrete EPSs and recruit other bacteria to thicken the biofilm (Rabin et al., 2015; Sharma et al., 2016). Aggregated bacteria synchronize their gene expression by a process called quorum sensing, by which the community regulates its cell density and performs some collective behaviors (Lyon and Muir, 2003; Sharma et al., 2016). A multispecies biofilm often forms an intricate micro-ecological system. Bacteria with different oxygen demands occupy distinct biofilm layers (Rabin et al., 2015). Synergistic and antagonistic interactions between species allow the biofilm to resist external disturbances (Oliveira et al., 2015; Del Pozo, 2017). A mature biofilm will sometimes undergo a process called dispersion, during which the bacteria detach from the biofilm and become planktonic. The outer environment influences the balance of the planktonic and biofilm state. Severe environments, such as high level of antimicrobial agents, favor communities to generate a biofilm. When the condition becomes more favorable, the bacteria are released by dispersion and become planktonic (Sharma et al., 2016). Biofilm formation often alters the physiological character of bacteria. The bacteria within the biofilm have a different gene expression profile and unique characteristics compared with their planktonic counterparts (Del Pozo, 2017; Dumitrache et al., 2017), and the EPSs of a biofilm also influence the behavior of communities (Rabin et al., 2015). It is rational to speculate that the state of bacteria also influences their adherent ability, but the details are still unclear.

### Other Possible Sperm Impairment Factors of Vaginal *Lactobacillus*

Vaginal *Lactobacillus* species exert anti-oxidant effects *via* a myriad of mechanisms that provide protection against the impact of ROS caused by immobilization factors. Barbonetti et al. showed that some *Lactobacillus* species can prevent the sperm immobilization effect of soluble factors produced by *E. coli*; a reduced ROS level is associated with this benefit (Barbonetti et al., 2013). LPS is absent in gram-positive bacteria; however, Peptidoglycan on the surface of bacteria has a toxic effect that is similar to LPS. It can recognize TLR2 presented on the acrosome surface, reduce the motility of human sperm and induce the apoptosis of spermatozoa (Fujita et al., 2011). Thus, the general sperm immobilization effect of vaginal *Lactobacillus* is controversial. Furthermore, genomic studies have revealed the hemolysin genes on all four *Lactobacillus* species that dominant vaginal communities, and some strains of *L. rhamnosus* and *L. fermentum*, also contain hemolysin genes (Makarova et al., 2006). It is still unclear whether these genes can be expressed and transported through the cell wall, and the potential immobilization effect of these *Lactobacillus* species must be determined by additional studies.

### Significance of Vaginal Lactobacillus to Fertility

A high *Lactobacillus* load can enhance the vitality of gametes in the male genital tract and stabilize the microenvironment of the female genital tract. These probiotic effects ultimately result in a positive effect on fertility (Rowe et al., 2020). However, the adherent effect of genital *Lactobacilli* can also induce a sperm impairment effect when there is a high bacterial load. Given that vaginal *Lactobacillus* species do not dramatically influence fertility, the sperm impairment effects of vaginal *Lactobacillus* should be relatively mild under physiological conditions compared with the powerful effects of urogenital pathogens. Spermatozoa with low motility or a vulnerable morphology will be readily agglutinated and immobilized, but those with higher motility and a stable morphology can overcome the weak affinity and remain intact. This mild impairment effect can perform a selection effect on ejaculated spermatozoa: spermatozoa with low motility and a vulnerable morphology will be weeded out in the vaginal tract. This foundation can be found in postcopulatory sexual selection of polyandrous wild animal species (Rowe et al., 2020). Vaginal *Lactobacillus* species, combined with other selection mechanisms in the female genital system, eliminate these abnormal spermatozoa and therefore ensure the quality of the paternal genome. However, this mild sperm impairment may also be responsible for some cases of unexplained infertility. For women whose partners are subfertile, this selection mechanism may exert a negative effect. Given that their partners are potential patients with oligospermia, asthenospermia, or teratospermia, the sperm selection effect of vaginal *Lactobacillus* species may amplify the condition. In addition, vaginal *Lactobacillus* species may show a powerful sperm impairment effect if they are present at a very high concentration. Probiotic therapies with vaginal administration may induce the overload of vaginal *Lactobacillus.* The concentration of vaginal *Lactobacillus* species fluctuates along with temporal and individual differences, and thus the sperm impairment effect of vaginal *Lactobacillus* species may result in variable consequences in different hosts. Furthermore, bacteria within a biofilm may present distinct adherence profiles compared with their planktonic counterparts. Biofilm ESPs may provide excessive interactions between spermatozoa and communities, a phenomenon that is often ignored in the co-incubation test. Some species have a strong tendency to form a biofilm *in vivo*, and the adherent effect of biofilm may play a major role instead of the planktonic counterparts (Ventolini et al., 2015). The sperm impairment effect of vaginal *Lactobacillus* species may also influence the evolution of reproductive strategies. As vaginal *Lactobacillus* species only dominate vaginal communities in humans, the sperm selection of vaginal *Lactobacillus* species may denote some unique characteristics in human reproductive strategies. Understanding this interaction is also essential for infertility treatment. With the popularization of probiotic drugs for vaginitis treatment, this interaction should be extensively investigated, and the adverse effect of vaginal probiotic drugs should be reconsidered. Despite their medicinal value, related research findings may also give new contraceptive strategies. Studies of the sperm–*E. coli* interaction have demonstrated the possibility of contraceptive vaccines. This strategy has been demonstrated as anti-sperm receptor antibodies; previous contraceptive vaccines mimic the antibodies from *E. coli* and have proven its efficacy. Additional studies of vaginal *Lactobacillus* species can provide new choices for contraceptive vaccines (Kaur et al., 2015).

## Conclusion

Lactobacillus are common bacteria in the vaginal tract of healthy females. While in previous studies authors had usually focused on their probiotic effect, in this review we provided a new perspective on how vaginal Lactobacillus impair the ejaculated spermatozoa, which contradicts the original view of genital Lactobacillus as a probiotics. The key point is the concentrations of vaginal Lactobacillus are different from that of seminal Lactobacillus and what is reported *in vitro* tests. With a periodic change in nutrition supplementation, vaginal Lactobacillus present a huge temporal difference in concentration and distribution, which is often ignored but may influence the fertility in a subtle way, and the marked individual differences in the vaginal microbiome in asymptomatic females also underlies variable effects. Therefore, while the probiotic effects of genital Lactobacilli have been widely demonstrated, it remains uncertain whether these Lactobacillus species play a positive role in fertilization, as the generally supposed probiotic characteristics may induce variable results under different bacterial concentrations. With a deeper understanding of microbiology, the boundary of probiotics and pathogens has become indistinct and controversial. For each probiotic with potential commercial value, it is of great importance to evaluate its potential pathogenic elements from different fields’ points of view. Given that these potential elements may induce complications during probiotic therapies and partially elucidate unexplained infertility, extensive study is essential.

## Author Contributions

TC designed the review. FZ searched for the materials. TC, FZ, and JD analyzed the references and wrote the manuscript. All authors discussed the results and commented on the manuscript.

## Funding

This work was supported by grants from the National Natural Science Foundation of China (No. 82060638) and “double 10-thousand plan” of Jiangxi Province (innovation and technology professionals as the high-end talent).

## Conflict of Interest

The authors declare that the research was conducted in the absence of any commercial or financial relationships that could be construed as a potential conflict of interest.
